# Reducing Health Disparities and Improving Health Equity in Saint Lucia

**DOI:** 10.3390/ijerph13010037

**Published:** 2015-12-22

**Authors:** Kisha Holden, Lisa Charles, Stephen King, Brian McGregor, David Satcher, Allyson Belton

**Affiliations:** 1 Satcher Health Leadership Institute, Morehouse School of Medicine, 720 Westview Drive SW, Atlanta, GA 30310, USA; bmcgregor@msm.edu (B.M.); dsatcher@msm.edu (D.S.); abelton@msm.edu (A.B.); 2 Victoria Hospital, Hospital Road, Castries P.O. Box 1750, St. Lucia; lisa_charles@hotmail.com; 3 Tapion Hospital, Tapion Road, Castries P.O. Box 1780, St. Lucia; kings@candw.lc

**Keywords:** St. Lucia, diabetes, hypertension, health equity

## Abstract

St. Lucia is an island nation in the Eastern Caribbean, with a population of 179,000 people, where chronic health conditions, such as hypertension and diabetes, are significant. The purpose of this pilot study is to create a model for community health education, tracking, and monitoring of these health conditions, research training, and policy interventions in St. Lucia, which may apply to other Caribbean populations, including those in the U.S. This paper reports on phase one of the study, which utilized a mixed method analytic approach. Adult clients at risk for, or diagnosed with, diabetes (*n* = 157), and health care providers/clinic administrators (*n* = 42), were recruited from five healthcare facilities in St. Lucia to assess their views on health status, health services, and improving health equity. Preliminary content analyses indicated that patients and providers acknowledge the relatively high prevalence of diabetes and other chronic illnesses, recognize the impact that socioeconomic status has on health outcomes, and desire improved access to healthcare and improvements to healthcare infrastructures. These findings could inform strategies, such as community education and workforce development, which may help improve health outcomes among St. Lucians with chronic health conditions, and inform similar efforts among other selected populations.

## 1. Introduction

St. Lucia is an island country located in the Eastern Caribbean, with an approximate population of 179,000, where the life expectancy (of the total population) is 77.41 years. St. Lucia has one of the highest diabetes-related mortality rates [1]. Unfortunately, health indicators applicable to other Caribbean countries, many with larger populations, reveal similar challenges. Haiti is an island country located in the Western Caribbean, with an estimated population of 9,996,731, and has a life expectancy of 63.18 years. Disaster-related health complications and stroke are the top two causes of death, while diabetes ranks seventh [2]. Jamaica, also located in the Western Caribbean, has an estimated population of 2,930,050 and a life expectancy of 73.48 years. Chronic, non-communicable diseases, such as hypertension, cancer, and diabetes, account for the most common causes of death [2]. According to a report by the Caribbean Commission on Health and Development (2006), the four leading causes of death are heart disease, cancer, stroke, and diabetes, which mirrors the ranking order of mortality causes in St. Lucia. Socioeconomic, environmental, and lifestyle or behavioral factors underlie most of the causes of preventable diseases, injury, and death, and avoidable health costs in the Caribbean. Moreover, current limitations to improving community health outcomes in the Caribbean include challenges with increasing the human resource base, health leadership capacities, and deficiencies in management systems.

Diabetes Mellitus (specifically Type II) is among the leading causes of death in St. Lucia, which has one of the highest diabetes related mortality rates in the world (14.37% of total deaths, 79.22 per 100,000) [1,3]. However, the body of scientific literature on the exact rates of morbidity and mortality associated with diabetes in St. Lucia is limited due to the lack of available data. Thus, the information provided is based on the best available science, which further substantiates the need for more targeted research with this population. A global burden of disease profile indicates that the top three risk factors that account for the most disease burden in St. Lucia is poor diet, high body-mass index, and high fasting plasma glucose [4]. These issues have particular relevance for vulnerable and high-risk populations, including poor, homeless individuals, and people of African descent in specific locations around the globe. Although there are important differences between various Caribbean populations, delving into the unique risk and protective factors of a small sample of the population in St. Lucia may yield useful data and relevant information for interventions aimed at selected vulnerable populations in the U.S., including Black Caribbean immigrants and some African Americans.

Increasing knowledge and awareness of chronic health conditions and strategies to reduce associated morbidity and mortality in St. Lucia and other Caribbean nations may inform prevention and intervention efforts among Caribbean immigrants residing in the United States. Diabetes and other chronic non-communicable diseases, such as hypertension and cardiovascular disease, and associated risk factors, such as obesity, are also challenges among this population. Some evidence indicates that rates of prevalence and incidence of overweight and obesity experienced by Caribbean immigrants are among the highest observed in comparison to other immigrant subgroups [5]. Further, little is known about effective approaches to address these health disparities among Caribbean populations, perhaps due in part to sparse attention paid to cultural differences among Afro-Caribbeans and African Americans within health related research efforts [6,7]. These challenges require scientific application and documentation of population health interventions designed to reduce diabetes mortality and morbidity in the U.S. and the Caribbean. Available health indicators demonstrate that St. Lucians have a similar health profile to Black Caribbeans and some African Americans and investigating innovative models of prevention and intervention to strengthen individuals and families at increased risk for obesity and diabetes in St. Lucia may help guide efforts aimed at these populations in the U.S.

Addressing the multi-faceted health needs of St. Lucia populations is a complex issue that warrants attention from policymakers, clinicians, researchers, scientists, and public health professionals that can offer unique perspectives and strategies to support efforts for greater well-being among individuals. There is growing diversity among these nations and significant changes in the constellation of risk factors that influence health and health outcomes. Therefore, it is imperative to examine evidence-based models, develop strategic health policies, implement effective community-based programs, and engage in innovative research aimed at improving individuals’ longevity and quality of life in St. Lucia. These research efforts can subsequently enhance diabetes reduction initiatives among other Caribbean nations and Caribbean immigrants in the U.S.

The specific aim of the proposed research investigation is to design a strategic and focused implementation plan for community health education and prevention approaches, research workforce development related to the training of local investigators in conducting medical and behavioral research and developing research interventions. This paper reports on phase one of this aim, utilizing qualitative research methods to gather valuable data on challenges and opportunities to address access and quality of health care, the life experiences of individuals living with chronic health conditions, and practice and policy strategies that may be useful in improving health outcomes and health equity in St. Lucia.

## 2. Methods

### 2.1. Sample

Adult men and women who were patients at five private or public healthcare facilities in St. Lucia were recruited to provide their perspectives on health care quality and the health status of St. Lucians. St. Lucian health care providers at each of the five sites, and two local health educators recruited individuals who were: (1) receiving health care services at each site, and (2) were at risk for, or diagnosed with, diabetes. Additionally, health care providers/clinic administrators were recruited to provide information on the health status of St. Lucians, health care services, challenges, and opportunities of chronic health conditions among St. Lucians. Study investigators recruited health care providers who provide health care for individuals diagnosed with diabetes, or are in a high-risk group. This study was approved by the Morehouse School of Medicine Institutional Review Board and the St. Lucia Medical and Dental Council Ethics Research Committee to ensure that the protection of human subjects and ethical practice standards of research for the investigation were achieved.

### 2.2. Study Sites

The private health care facilities that were study sites were the St. Lucia Diabetes and Hypertension Association (Castries, St. Lucia), Tapion Hospital (Castries, St. Lucia), and St. Anthony’s Medical Centre (Vieux Fort, St. Lucia). The public health care facilities that were study sites were Victoria Hospital (Castries, St. Lucia) and St. Jude Hospital (Vieux Fort, St. Lucia).

### 2.3. Measures

A client needs measure was developed by the research team, in collaboration with St. Lucia health care providers and administrators; and assessed demographic/background information including age, gender, race, primary language, ethnicity, marital status, employment status, household income, health insurance status, and education level. The measure asked participants to rank their primary health concerns, based on a list of conditions/issues that included items such as mental health, overweight/obesity, drug/alcohol abuse, diabetes, and heart disease/hypertension. The measure also asked participants to rank their social and economic resource concerns including items, such as food and shelter, transportation, employment and job training, and public safety. Respondents were also queried via a series of questions on their overall satisfaction of the health care services they receive using a Likert scale of 1–5, with 1 indicating *Strongly Disagree* and 5 indicating *Strongly Agree.* Finally, respondents were asked about their personal history of tobacco use, as well as history of participation in health educational classes/workshops, and overall relationship with their healthcare provider.

### 2.4. Chronic Disease Management Measure

A provider measure was developed by the research team, in collaboration with St. Lucia health care providers and administrators. The measure collected demographic background information including age, gender, race/ethnicity, primary spoken language, length of time in practice, current area of expertise, and current practice base. The questionnaire included a series of questions regarding the reported amount, by percentage, of collaborative care/services that their patients received, as well as the treatment needs (in percentage) by condition of their patients. They were also queried on their level of educational interaction (in percentage) with their respective patient populations by the condition of their patients. Finally, respondents were asked to rank the top three (3) needs that are most urgent in improving their respective patients’ healthcare (referrals to specialty care; improved capacity for data gathering, monitoring, and tracking; better community engagement; *etc.*). There was also an open-ended question asking providers to indicate important health care needs in St. Lucia.

### 2.5. Patient Recruitment

Patients were recruited prior to the study team visit by a local health educator and health care staff at each of the five study sites. Interested individuals were scheduled by site and participated in the informed consent process with trained research team members, with assistance by local health educators. Among the 157 men and women who participated in the study, 134 provided survey data. After providing informed consent, patients participated in a one-hour focus group, facilitated by a trained research team member. Questions from the focus group included concepts of chronic disease, thoughts of healthcare treatment, and the Social Determinants of Health. Afterwards, they completed the self-report assessment measure, administered by the trained research team members, which took approximately 20 min to complete. Each of the research participants received $15.00 USD/ECD 40.00 as incentive for study participation. No further contact was made with the research participants.

### 2.6. Providers/Clinic Administrator Recruitment

These individuals were recruited by research team members. Twenty-three interested individuals met one-on-one with a study team member, participated in the informed consent process, completed the Provider/Administrator survey (approximately 10 min), and participated in a 30-min key informant interview with the study team member. Questions from the key informant interview focused on healthcare treatment, treatment challenges, and suggestions of how to improve health and healthcare services. Participants at one site were recruited during an educational session, participated in the informed consent process, and only completed the survey. Participants were not compensated and no additional contact was made with the research participant. Forty-two providers/administrators completed surveys.

### 2.7. Data Analysis Plan

In this paper, basic descriptive statistics, such as mean, median, standard deviation, and percentages, were utilized to summarize the patient and health provider/administrator samples. Additionally, preliminary content analyses from the transcripts were conducted to ascertain patterns of themes, similarities, and differences. The full analysis of these qualitative data will include the formation of a coding team that will work to develop a coding schema, which is both relevant to the project and considers the types of data to be analyzed (*i.e*., transcripts), as well as different aspects of the data. Coding schemas may consider the structure of the interview, the salient categories, and concepts that inform current theories about the phenomena or group, and themes that emerge through close reading of the texts. All textual materials will be analyzed with these techniques. This will allow researchers to discover themes and patterns in qualitative data during the entire process of the research. Theoretical saturation will occur when no new patterns or themes emerge in the data. An explanatory model will then be developed that will inform prevention, education, and intervention strategies.

## 3. Results

### 3.1. Patient Characteristics and Health Behaviors

Table 1 summarizes the patients included in the dataset. Among the 124 patients who reported their gender, 73.1% (*n* = 98) identified as female and 19.4% (*n* = 26), identified as male; 10 participants did not report their gender. The majority of the patients (*n* = 119, 92.9%) identified as being of Black/African descent; the remainder of the patients providing race/ethnicity data identified as African-European (*n* = 4, 3.2%), East Indian (*n* = 2, 1.6%) and other (*n* = 3, 2.3%). Regarding marital status, 42.5% (*n* = 57) of the respondents indicated that they were married, while 38.8% (*n* = 52) indicated their marital status as single. Slightly over half of the responding patients, 53.8% (*n* = 70) indicated that they had four or more children; the next most frequent response was none (*n* = 18, 13.8%). The majority of the patients (*n* = 58, 40.8%) reported that they were unemployed. Twenty-eight (19.8%) respondents indicated that they were retired, and 25 patients (17.7%) reported that they were full-time students. These were the most frequent responses to the employment status question. Sixty-eight patients (53.6%) indicated that primary school was the highest level of education they had achieved, followed by some secondary school (*n* = 20, 15.7%). The annual household income of 40.5% (*n* = 45) of responding patients was under $2000. Seventeen patients (15.3%) indicated an annual household income in the range of $10,000–$14,999. A majority of the patients indicated that they were uninsured (*n* = 76, 59.8%), while 22% (*n* = 28) of the patients reported having National Insurance, which is provided by the St. Lucian Ministry of Health. Based on the survey results, the top three ranked service concerns (in order of ranking) are: health care (52.9%), crime/public safety (29.1%), and care for older persons (23.8%).

Based on the survey results, the top three ranked health concerns (in order of ranking) are: diabetes (92.5%), hypertension (53.7%), and heart disease (15.7%). The majority of the patients (*n* = 61, 45.5%) self-reported English as their primary language, closely followed by those who indicated both English and Creole/Patois as their primary language (*n* = 53, 39.6%).

**Table 1:** Patient demographics and reported health behaviors.

	Male	Female	Gender Not Reported	% of Total Sample’s Affirmative Responses	Total
**Race/Ethnicity**	*n* = 26	*n* = 93	*n* = 9		N = 128
Black/African	22	88	9	92.9	119
African-European	2	2	0	3.2	4
East Indian	1	1	0	1.6	2
Other	1	2	0	2.3	3
**Marital Status**	*n* = 26	*n* = 98	*n* = 10		N = 134
Single	7	43	2	38.8	52
Married	17	35	5	42.5	57
Divorced	0	2	1	2.3	3
Widowed	1	11	1	9.7	13
Common Law	1	7	1	6.7	9
**Number of Children**	*n* = 25	*n* = 96	*n* = 9		N = 130
None	3	14	1	13.8	18
One	3	9	1	10.0	13
Two	4	11	0	11.5	15
Three	2	12	1	11.5	15
Four or More	13	50	6	53.8	70
**Employment Status**	*n* = 28 *	*n* = 104 *	*n* = 10		N = 142
Unemployed	9	44	5	40.8	58
Part-Time	3	5	2	7.0	10
Full-Time	3	19	3	17.7	25
Student	0	2	0	1.4	2
Temporary	0	2	0	1.4	2
Homemaker	0	14	0	9.8	14
Retired	12	16	0	19.8	28
Disabled	1	2	0	2.1	3
**Highest Level of Education**	*n* = 23	*n* = 94	*n* = 10		N = 127
No Schoo1	3	3	0	4.7	6
Primary School	7	53	8	53.6	68
Some Secondary School	7	13	0	15.7	20
Secondary School Graduate	4	10	1	11.8	15
Technical or Vocational	2	0	0	1.7	2
Some College	0	6	0	4.7	6
College Graduate	0	3	0	2.3	3
Post Graduate	0	6	1	5.5	7
**Annual Household Income**	*n* = 19	*n* = 85	*n* = 7		N=111
Under $2000	3	39	3	40.5	45
$2000–$4999	2	10	3	13.5	15
$5000–$9999	3	6	0	8.1	9
$10,000–$14,999	4	13	0	15.3	17
$15,000–$24,999	3	5	0	7.2	8
$25,000–$34,999	2	3	0	4.5	5
$35,000–$49,999	1	5	0	5.4	6
$50,000 or More	1	4	1	5.4	6
**Health Insurance Coverage**	*n* = 24	*n* = 95	*n* = 8		N = 127
Private	2	14	0	12.6	16
National Insurance	9	16	3	22.0	28
Uninsured	12	60	4	59.8	76
Other Insurance	0	3	0	2.4	3
Two or More Types	1	2	1	3.1	4
**Primary Language**	*n* = 26	*n* = 98	*n* = 10		N = 134
English	15	41	5	45.5	61
Creole/Patois	5	13	1	14.2	19
Other	0	1	0	0.8	1
English and Creole/Patois	6	43	4	39.6	53
**Indicated Health Concerns**	*n* = 26	*n* = 98	*n* = 10		
Mental Health	1	5	0		6
Overweight	1	13	2		16
Drugs and Alcohol	2	4	0		6
Diabetes	23	92	9	92.5	124
Heart Disease	5	15	1	15.7	21
Dental	3	12	1		16
Reproductive Health	1	3	0		4
Quitting Smoking	0	3	1		4
Hypertension	15	49	8	53.7	72
HIV/STD	2	1	0		3
Other Concern 1	2	8	1		11
Other Concern 2	1	7	0		8
**Current Tobacco User**	*n* = 24	*n* = 97	*n* = 9		N = 130
Yes	0	4	1	3.8	5
No	24	93	8	96.2	125

* 6 respondents did not report their ethnicity.

### 3.2. Patient Focus Groups

A total of 14 focus groups were conducted, with 157 patients participating. Preliminary analyses of focus group data indicate that health care patients, health care providers, and health care administrators in St. Lucia recognize that chronic diseases, particularly hypertension, diabetes, and depression are widespread and often poorly managed in St. Lucia. Many participants also acknowledged that individuals with a lower socioeconomic status likely experience reduced health care access, poorer quality of care and poorer medical and behavioral health outcomes. However, participants also acknowledged that anyone, regardless of SES, may be diagnosed with a chronic disease. Additionally, participants indicated desire for better accessibility to healthcare services and improvements to existing healthcare infrastructures to provide better services. The relatively recent government policy to provide medications for diabetes and hypertension at no cost to patients diagnosed with such conditions was seen as beneficial to many patients; however, some changes are suggested as necessary to improve its overall positive impact.

### 3.3. Provider/Administrator Background and Chronic Disease Management

Table 2 summarizes information on the providers/administrators in the dataset. Of the 42 providers who completed the Health Care Provider/Administrator Survey, 81.0% (*n* = 34) identified as female, while 16.7% (*n* = 7) identified as male; only one (1) individual did not report gender (Table 2). The majority of the providers (*n* = 30, 71.4%) identified as being of African/Black descent; the remainder of the providers identified as African-European (*n* = 3, 7.1%) and other (*n* = 3, 7.1%). Six providers within the sample did not report their ethnicity. The majority of the providers surveyed (*n* = 17, 40.5%) reported nursing as their current area of expertise. There were six providers who reported that they were either junior physicians or general practitioner physicians (14.3%), while others reported as a consultant (*n* = 11, 26.2%) or an allied health professional (*n* = 8, 19.1%). Overall, the providers who completed the survey self-reported many years of experience. Of the 42 providers, over half indicated that they had been in the health professions for over 10 years (*n* = 23, 54.7%). Specifically, 38.1% (*n* = 16) of the overall sample had indicated that they had over 20 years of work experience in the health professions. The majority of the providers (*n* = 33, 78.6%) self-reported English as their primary language, while two providers (4.8%) self-reported English and Creole/Patois as their primary language. Only one provider stated that he/she spoke three primary languages, including English and Creole/Patois. Six providers within the sample did not report their primary language.

**Table 2:** Provider demographics and chronic disease management.

	Total	
	*n* = 42	%
**Gender**		
Male	7	16.7
Female	34	81.0
Unknown/Not Reported	1	2.4
**N**	42	
**Race/Ethnicity**		
African/Black	30	71.4
African-European	3	7.1
East Indian	0	0.0
Other	3	7.1
Unknown/Not Reported	6	14.3
**N**	42	
**Primary Language**		
English	33	78.6
Creole/Patois	0	0.0
English and Creole/Patois	2	4.8
>3 Languages	1	2.4
Unknown/Not Reported	6	14.3
**N**	42	
**Practice Experience**		
<1 year	5	11.9
1–5 years	9	21.4
6–10 years	5	11.9
11–15 years	3	7.1
15–20 years	4	9.5
>20 years	16	38.1
**N**	42	
**Area of Expertise**		
Nurse	17	40.5
Junior Physician	4	9.5
General Practitioner Physician	2	4.8
Consultant	11	26.2
Health Educator	0	0.0
Dietician	2	4.8
Other	6	14.3
**N**	42	
**Current Practice Base**		
Community Based Clinic	0	0.0
Hospital	32	76.2
Mental Wellness Centre	0	0.0
Private Practice	4	9.5
Administration	1	2.4
Public/Private Mix	0	0.0
Other	1	2.4
Multiple Settings	4	9.5
**N**	42	
**% of Patients with Depression Treatment Needs**		
0%–20%	11	26.2
21%–40%	18	42.9
41%–60%	8	19.0
61%–80%	2	4.8
81%–100%	0	0.0
No Response	3	7.1
**N**	42	
**% of Patients with Diabetes Treatment Needs**		
0%–20%	0	0.0
21%–40%	2	4.8
41%–60%	19	45.2
61%–80%	11	26.2
81%–100%	9	21.4
No Response	1	2.4
**N**	42	
**% of Patients with Hypertension Treatment Needs**		
0%–20%	1	2.4
21%–40%	2	4.8
41%–60%	16	38.1
61%–80%	14	33.3
81%–100%	8	19.0
No Response	1	2.4
**N**	42	
**% of Patients with Cardiovascular Disease Treatment Needs**		
0%–20%	2	4.8
21%–40%	8	19.0
41%–60%	17	40.5
61%–80%	8	19.0
81%–100%	4	9.5
No Response	3	7.1
**N**	42	
**% of Patients with Cancer Treatment Needs**		
0%–20%	18	42.9
21%–40%	4	9.5
41%–60%	11	26.2
61%–80%	5	11.9
81%–100%	1	2.4
No Response	3	7.1
**N**	42	
**% of Patients with STDs/HIV Treatment Needs**		
0%–20%	23	54.8
21%–40%	8	19.0
41%–60%	1	2.4
61%–80%	4	9.5
81%–100%	3	7.1
No Response	3	7.1
**N**	42	
**% of Patients with Substance Abuse Treatment Needs**		
0%–20%	25	59.5
21%–40%	10	23.8
41%–60%	3	7.1
61%–80%	2	4.8
81%–100%	1	2.4
No Response	1	2.4
**N**	42	
**Provider Needs (Top 3 Selected)**		
Increased Access to Patient Education Resources	28	66.7
Improved Public Health Policy	25	59.5
Enhanced Social Support	20	47.6

Note: **N** = Number of providers/administrators endorsing each response.

The majority of the providers (*n* = 32, 76.2%) reported a hospital as their current practice base. Other either reported working in a private practice (*n* = 4, 9.5%), multiple settings (*n* = 4, 9.5%), or in other areas, including administration (*n* = 2, 4.8%). Based on the survey results, the top three reported provider needs for improving the health care of their patients are: Increased Access to Patient Education Resources (66.7%), Improved Public Health Policy (59.5%), and Enhanced Social Support (47.6%).

### 3.4. Key Informant Interviews

Preliminary analyses of data from 23 key informant interviews revealed that providers view health policy reform as critically important in improving health outcomes of St. Lucians, but this requires commitment, political will, and strong leadership to effect change. Many providers felt strongly that patient education was key in improving the impact of prevention and treatment strategies aimed at those with chronic disease. Lastly, providers also indicated a need to increase the number of providers on the island to address healthcare workforce shortage and provide continuous medical education to strengthen the existing workforce.

## 4. Discussion

Our investigation’s preliminary analyses suggest that social determinants of health that impact vulnerable populations in the United States play a similar role in impacting health in in St. Lucia. For example, in the current study, there were considerably more women (73.1%) than men (19.4%) included in the sample, which is likely a reflection of the presence and availability of men engaged in each of the five health care sites where recruitment took place. The low participation rate of African American men in health related research, and clinical trials in particular, is well documented and is often discussed as a barrier to improving their health status [8]. Some evidence suggests that African American men are more likely to participate in health related research when encouraged by their doctors [9,10]. This should also be a recruitment goal when engaging Black Caribbean immigrants in the U.S. for research investigations. This resonates with the recruitment efforts for this study as it was noted that St. Lucian nurses were critically important to engaging potential research participants and achieving the sample size that was obtained. Future research initiatives would do well to consider the impact patient-physician relationships have on engaging St. Lucian men, in an effort to develop a better understanding about their health care behaviors, which is key to developing successful prevention and intervention strategies.

It was also observed that over half of the survey respondents (53.8%) reported having four or more children, which should be considered when developing interventions with this population. Health care strategies aimed at parents that are family centered and designed to engage the family unit are vital to success. These strategies may include designing health promotion and prevention activities for children that might involve the school system and educating parents to deliver positive health care messages and model healthy behaviors for their children. Additionally, recruiting parents for research engagement should consider that securing childcare needs may improve availability and motivation to participate.

Results of the preliminary analyses of demographic data and focus group discussions indicate that the social determinants of health greatly impact these patients’ access to care and their attitudes on improving health care outcomes. Demographic data reflect that a significant percentage of patients were unemployed (58%), had an annual household income of under $2000 (40.5%), attained a primary school education as their highest level of learning (53.6%), and were uninsured (59%). Patients experiencing these challenges have firsthand knowledge of the difficulties that are associated with a lower SES including reduced health care access, poorer quality of care, and poorer medical and behavioral health outcomes, all of which emerged as a theme in the focus group data. This is an important finding for practice and policy strategists to consider when developing interventions suggesting a relationship between quality of life and improving health care outcomes.

A review of survey and key informant interview data from providers and one administrator reveal interesting conclusions that mirror many of the findings observed in the analyses of patient data. Interestingly, a slight majority of the providers (54.7%) who completed surveys indicated that they had been in the health professions for over 10 years and 38.1% have over 20 years of work experience in the health professions. This is a pilot study and reflects a small sample of the health care workforce in St. Lucia. However, it is important to note that their perspectives on the health care challenges facing St. Lucians and their experiences with providing chronic disease management are informed by health care professionals with considerable experience in practice. Thus, they have been able to observe patterns and trends in patient health behaviors, patient attitudes and the impact of the social determinants of health in St. Lucia over a relatively long period of time, as compared to early career health care professionals.

One area where patients and providers/administrators do not appear to have similar views is in regards to the importance of addressing the mental and behavioral health needs of St. Lucians. While only 4% of patients who completed the survey indicated mental health as a concern, 42.9% of providers reported that up to 40% of their patients need depression treatment. While extensive work is needed to efficiently collect epidemiological data concerning mental health among St. Lucians, improvements in the mental health care system are vital to meeting existing behavioral health needs [11,12]. There is also the challenge of immigrant subgroups being described as African American within research studies, and are not uniquely targeted, which masks important differences. The mental health risk profile of Black Caribbean immigrants in the U.S. is considerably diverse. Factors, such as age at immigration, native language, and foreign birth, mitigate the risk and cultural considerations within the U.S. health care system likely affect treatment outcomes [13].

The role of mental health stigma, which was a topic of discussion in patient focus groups and key informant interviews, may have an impact on patients’ willingness to discuss and acknowledge mental health concerns they have for themselves and for the communities where they live. Stigma has also been recognized as a barrier for improving mental health engagement among African Americans [13,14]. It may also be the case that patients were not prepared to have a discussion about mental health. While patients were informed that mental health conditions such as depression would be among the chronic health conditions that would be discussed, emphasis was placed on illnesses such as diabetes, hypertension and heart disease. During several focus groups, there was a period of silence immediately following the facilitator posing questions about mental health and mental illness. Strategies to introduce the topic of mental health among patients to obtain valuable data that may guide intervention approaches should be explored further.

Providers indicated that increased access to patient education resources, improved public health policy and enhanced social support were the top three reported provider needs. This is commensurate with key informant interview data indicating that providers believe that health policy reform is important improving health outcomes of St. Lucians but requires commitment, political will and strong leadership to effect change. Additionally, many providers felt strongly that patient education and more social support was critical to improving the impact of prevention and treatment strategies aimed at those with chronic disease(s). These results suggest that among the health care professionals we surveyed, there is strong support for a comprehensive approach to addressing the health care needs of St. Lucians with chronic health conditions which includes patient engagement, policy strategies and practice improvements.

It is our intention to use these data to assess the efficacy of a model that is devised based on study findings. Overall, results of this preliminary analysis suggest that improving health outcomes among St. Lucians may include strengthening patient education, addressing the social determinants of health, improving public health policy, reducing mental health stigma, and developing family centered approaches for healthcare engagement are key considerations. Perhaps culturally-sensitive, community-based prevention program aimed at individuals, families, and communities that is based on informing St. Lucians about selected target health conditions, including signs and symptoms, risk and protective factors, and strategies to prevent onset and impede the progression of these illnesses once the disease process has begun will have success.

The impact of interventions aimed at reducing health disparities prevalent among St. Lucians could form a basis for targeting Caribbean immigrants experiencing similar problems in the United States and for informing policies aimed at reducing disparities in health. Increased understanding of cultural similarities and differences between Caribbean immigrants in the United States and St. Lucian populations may be valuable in increasing the effectiveness of intervention programs, policies and research investigations aimed at reducing medical health and mental health disparities among vulnerable and high risk populations. It is imperative that multi-dimensional interventions be designed, implemented and evaluated that includes contributions from: (1) diverse health professionals and community health educators; (2) government entities concerning health, education, and economic development; (3) academic institutions; and (4) health policy advocacy organizations. The collective strengths of these entities can establish a strong foundation to build upon to promote community health and reduce disparities.

There are several limitations to this preliminary study; and our next steps include: (1) refining and increasing the sample size for another phase of investigation to further support the diversity and generalizability of study findings; (2) establishing a novel approach to engaging urban and rural populations in order to better understand the distinctions of these populations; and (3) implementation of a pilot intervention based on the results of the preliminary study. The authors acknowledge that there is a need for future investigations concerning this important topic of scientific inquiry.
